# Prevalence of self-medication and associated factors among female students of health science colleges at Majmaah University: A cross-sectional study

**DOI:** 10.3389/fpubh.2023.1090021

**Published:** 2023-02-16

**Authors:** Shamshad Begum Loni, Raed Eid Alzahrani, Mansour Alzahrani, Mohammad Owais Khan, Rafia Khatoon, Huda Hakim Abdelrahman, Zeinab A. Abd-Elhaleem, Munira Mohammed Alhaidari

**Affiliations:** ^1^Department of Basic Medical Science, College of Medicine, Majmaah University, Al Majmaah, Saudi Arabia; ^2^Department of Family and Community Medicine, College of Medicine, Majmaah University, Al Majmaah, Saudi Arabia; ^3^Department of Aeronautical Engineering, Shri Devi Institute of Technology, Mangalore, India; ^4^Department of Forensic Medicine and Toxicology, College of Medicine, Ain Shams University, Cairo, Egypt; ^5^College of Medicine, Majmaah University, Al Majmaah, Saudi Arabia

**Keywords:** quick relief, laziness, antispasmodics, adverse effect, health science colleges, self-medication, source of medication

## Abstract

**Introduction:**

Globally, the prevalence of self-medication among young people has increased exponentially. Due to the basic knowledge and easy access to medicines, undergraduate students at health science colleges are likely to self-medicate. This research was undertaken to assess self-medication prevalence and its contributing factors among female undergraduate students in health science colleges at Majmaah University, Saudi Arabia.

**Materials and methods:**

A descriptive, cross-sectional study involving 214 female students from the Majmaah University in Saudi Arabia's health science colleges—Medical: (82, 38.31%) and Applied Medical Science College (132, 61.68%)—was conducted. A self-administered questionnaire with sociodemographic information, drugs used, and reasons for self-medication was used for the survey. Non-probability sampling techniques were used to recruit participants.

**Results:**

Of the 214 female participants, 173, 80.84 % (medical: 82, 38.31% and applied medical science: 132, 61.68%) confirmed that they were on self-medication. The majority of participants (42.1%) were between the ages of 20 and 21.5 years (mean ± SD: 20.81 ± 1.4). The main reasons for self-medication were quick relief from the illness (77.5%) followed by saving time (76.3%), minor illnesses (71.1%), self-confidence (56.7%), and laziness (56.7%). The use of leftover drugs at home was common among applied medical science students (39.9%). The main indication for self-medication included menstrual problems (82.7 %), headache (79.8%), fever (72.8%), pain (71.1%), and stress (35.3%). The most common drugs used included antipyretic and analgesics (84.4%), antispasmodics (78.9%), antibiotics (76.9%), antacids (68.2%), multivitamins, and dietary supplements (66.5%). On the contrary, the least used drugs were antidepressants, anxiolytics, and sedatives (3.5, 5.8, and 7.5 %, respectively). Family members were the main source of information for self-medication (67.1%), followed by self-acquired knowledge (64.7%), social media (55.5%), and least were friends (31.2%). For adverse effects of the medication, the majority of them consulted the physician (85%) followed by consulting the pharmacist (56.7%) and switched to other drugs or decreased drug dosage. Quick relief, saving time, and minor illness were the main reasons for self-medication among health science college students. It is recommended to conduct awareness programs, workshops, and seminars to educate on the benefits and adverse effects of self-medication.

## 1. Introduction

To be in good health is an essential component of one's life, so having access to an efficient healthcare system in a community is important. Most of the common health ailments are treated by the individual themselves without medical supervision, termed self-medication (SM) (1). According to World Health Organization (WHO) definition, “Self-medication involves the use of medicinal products by the consumer to treat self-diagnosed disorders or symptoms, or the intermittent or continued use of medication prescribed by a physician for chronic or recurrent diseases or symptoms” (1, 2).

Self-medication (SM) is common in both developed and developing countries, with prevalence rates ranging from 25.6 to 73.6%. It is also associated with a positive perception of the country's healthcare system (2).

When practiced effectively, SM could benefit both the individual and the healthcare system by potentially saving lives in emergency situations, minimizing long waiting times for proper medical assistance, and also lowering healthcare costs. On the contrary, SM may increase risks such as unnecessary use of medication, extended duration of consumption, drug–drug interactions, and polypharmacy.

Self-medication increases the risk of using illegal drugs, developing drug dependence, and masking the underlying medical conditions, all of which can compromise human safety, leading to drug resistance, and making diagnosis more challenging (3, 4). According to the researchers, the use of over-the-counter medications is common and inappropriate among undergraduate students in universities around the world (3, 5, 6). Owing to the overwhelming use of social media, students are now relying more on the internet than healthcare professionals in their health-related information (4, 7, 8). As a result, more college students are practicing SM to treat self-diagnosed illnesses (9). SM has a greater impact on students with a medical knowledge background as they have easy access to information from their curriculum and literature (10). Previous studies on SM practice among medical students reported that the prevalence differed in developing and developed countries (6–11). Easy availability of physician samples from pharmaceutical representatives and the white coat effect of being health science students ensure trouble-free access to drugs from pharmacies (12). Studies have shown that the practice of SM is influenced by multiple factors such as education, family, friends, and the rule and regulations of that particular country. Medical textbooks, articles, and social media marketing strategies of pharmaceuticals persuade participants to contemplate purchasing medications without prescription rather than seeking the advice of a healthcare professional. Previous research studies have shown that the causes of SM include minor illnesses, previous experience in treating similar illnesses, financial concerns, and a scarcity of physicians. Medical textbooks, articles, and social media marketing strategies of pharmaceuticals persuade participants to contemplate purchasing medications without prescription rather than seeking the advice of a healthcare professional. Previous research studies have shown that the causes of SM include minor illnesses, previous experience treating similar illnesses, financial concerns, and a scarcity of physicians (13). However, there is a paucity of studies that show the prevalence of SM among undergraduate students of health science colleges in Majmaah, Saudi Arabia. Hence, the study was designed to assess the prevalence of self-medication practices, associated factors, and knowledge of adverse effects of drugs among female students of Medical and Applied medical science colleges at Majmaah University, Majmaah, Saudi Arabia. Thus, creating awareness of the benefits and adverse effects of SM and its impact on the health of the young generation in society.

## 2. Materials and methods

### 2.1. Study design and sample size

A descriptive cross-sectional study was conducted from September to November 2021. The study was designed using a simple random sampling technique. Female undergraduate students from level 1 to level 5 were grouped into medical and applied medical science student groups. The applied medical science group included students from Nursing, Physiotherapy, Radiology, and Medical laboratory colleges.

Using Raosoft^®^ software, the sample size of 278 was calculated taking 1,000 as the total female student population of health science colleges of Majmaah University, with a 5% margin of error, 95% confidence interval, and 50% response distribution. By adding 10% as the non-response rate, the sample size was increased from 278 to 306. Ten questionnaires were distributed among the female students. Only 290 students (93.6%) agreed to participate. However, 214 (73.8%) students submitted a completely filled questionnaire. Of 214 participants, 173, 80.84 % (medical: 82, 38.31% and applied medical science: 132, 61.68%) confirmed that they were on self-medication 3 months prior to the start of the survey. The majority of participants (42.1%) were between the age group 20 and 21.5 years (mean ± SD: 20.81 ± 1.4). To collect data, a verified 50-item self-administered questionnaire form with six domains: sociodemographic, the need for self-medication, indications, sources of knowledge, medications used, and attitude toward an adverse effect of drug reaction was used. Both English and Arabic versions of the questionnaire forms were used to avoid language-based bias. A pilot study was done to assess the validity of the questionnaire. The reliability of 81.9% was observed using Cronbach's alpha test. Before starting the procedure, participants were debriefed about the purpose of the study followed by providing written consent. A simple randomized study design was done.

### 2.2. Inclusion/exclusion criteria

All female students from medical and applied medical science colleges in the main building of Majmaah University willing to provide consent were included in the study. Those who were absent during the data collection period were excluded from the study. Students who were unable to submit the completed questionnaires were counted as non-respondents.

### 2.3. Ethical approval

The study was conducted in accordance with the Declaration of Helsinki, and approved by the Institutional Review Board of Majmaah University (MUREC-Dec.1 5/COM-2 0 20/13-1)-2020/174/021). The confidentiality of information was assured.

### 2.4. Data analysis

Microsoft Excel was used for data collection. Statistical software IBM^®^ SPSS Statistics, version 25 (IBM Corp., Armonk, N.Y., USA) was used to perform descriptive and inferential statistics. Descriptive statistics such as frequency, percentage (%), mean ± SD, and distribution (skewness and kurtosis) were used to present data using a significance level of *p* ≤ 0.05. Categorical variables were assessed using frequencies and percentages, through Univariate analysis. Analytical statistics were performed using odds ratio (OR), 95% confidence intervals (CIs), and logistic regression. Using cross-tabulation, the chi-square test was used to compare the variables between medical and applied medical college students with self-medication. The variables were correlated using Pearson correlation. Tables were used to summarize the findings.

## 3. Results

Of the 290 female participants who agreed to participate, 214 (73.8%) completed the survey questionnaire with a skewness of 0.66 and kurtosis of 0.16, which was submitted for data analysis. Preceding 3 months of the survey, 80.84% (*N* = 173) participants had self-medicated, while 19.16% (*N* = 41) did not self-medicate or (odds ratio): 1.43; 95% confidence interval (CI): 0.69–2.95; *P* = *0.33*). Most respondents (N = 90; 42.1%) were between 20 and 21.5 years old (mean ± SD, 20.81 ± 1.4). There was a statistically significant association between the SM and urban (*N* = 149, 86.6%) as a living area (OR=4.9; 95%CI=2.3–10.3; *P*<*0.0001*). Previous comorbid conditions documented among medical students (9.23%) were gastritis, allergy, otitis media, and pre-diabetic condition, while applied medical science students (5.6%) reported anemia, asthma, hyperthyroidism, and gallstones (Table 1).

**Table 1:** Socio-demographic distribution among female participants (*N* = 214).

**Self–medication 3 months preceding the research study**						
**Variables**	**Category**	**Total**	**Yes**	**No**	**Odds ratio (95% CI)**	***P-*** **value**
		**No. (%)**	**No. (%)**	**No. (%)**		
College	Medicine	82 (38.31)	69 (84.15)	13(15.86)	1.43 (0.69–2.95)	0.33
	Applied medical science	132 (61.68)	104(78.79)	28(21.21)		
	Total	214 (100)	173 (80.84)	41(19.16)		
Residence	Urban	172 (80.4)	149 (86.6)	23 (13.4)	4.9 (2.3–10.3)	< 0.000^**^
	Rural	42 (19.6)	18 (42.9)	24 (57.1)		
Lifestyle	Family	194 (91.1)	158 (81.44)	36(18.55)	1.46 (0.5–4.3)	0.48
	Friends /Alone	20 (9.4)	15 (75)	5 (23)		
Chronic Illness	Suffering	20 (9.35)	18 (90)	2 (10)	2.26 (0.5–10.17)	0.29
	Not suffering	194 (90.65)	155 (79.89)	39(20.10)		

** *P* ≤ 0.001 is highly significant.

The most common reasons for SM were quick relief from illnesses (*N* = 134, 77.5 %; *X*^2^ = 4.09, *P* = 0.04), followed by saving time (*N* = 132, 76.3 %; *X*^2^, 3.8 *P* = 0.05), self-confidence (*N* = 98, 56.7%; *X*^2^, 6.2, *P* = 0.013), and laziness to visit the physician (*N* = 98,56.7%; *X*^2^, 6.2, *P* =0.013), all of which were statistically significant among the participants. Considering the disease as a minor illness (*N* = 123, 71.1 %; *X*^2^, 1.8 *P* =. 18) among the participants was statistically non-significant. The use of leftover drugs kept at home was more common among the applied medical science students (*N* = 45, 43.2%) as compared to medical students (*N* = 24, 34.8%) (Table 2).

**Table 2:** Reasons for self-medication (*N* = 173).

**Reasons**	**College**	**Yes**		**No**		***X^2^***	***P*** **- value**
		**No**.	**%**	**No**.	**%**		
Time saving	Medicine	58	84.1	11	15.9	3.8	0.05^*^
	Applied medical science	74	71.2	30	28.9		
	Total	132	76.3	41	23.7		
Illness as minor	Medicine	53	76.8	16	23.2	1.8	0.18
	Applied medical science	70	67.3	34	32.7		
	Total	123	71.1	50	28.9		
Economical	Medicine	21	12.1	48	27.7	1.91	0.17
	Applied medical science	22	12.7	82	47.4		
	Total	43	24.9	130	75.1		
Quick relief	Medicine	48	69.6	21	30.4	4.09	0.04^*^
	Applied medical science	86	82.7	18	17.3		
	Total	134	77.5	39	22.5		
Previous experience	Medicine	47	68.1	22	31.9	0.25	0.61
	Applied medical science	67	64.4	37	35.6		
	Total	114	65.9	59	34.1		
Easy availability of drugs	Medicine	35	50.7	34	49.3	0.008	0.93
	Applied medical science	52	50	52	50		
	Total	87	50.3	86	49.7		
Free physician sample	Medicine	16	23.2	53	76.8	0.03	0.87
	Applied medical science	23	22.1	81	77.9		
	Total	39	22.5	134	77.5		
Self-confidence	Medicine	47	68.1	22	31.9	6.2	0.013^*^
	Applied medical science	51	49.0	53	51.0		
	Total	98	56.7	75	43.3		
Laziness to go to doctor/physician	Medicine	47	68.1	22	31.9	6.2	0.013^*^
	Applied medical science	51	29.5	53	30.6		
	Total	98	56.7	75	43.3		
Use of leftover drugs kept at home	Medicine	24	34.8	45	65.2	1.3	0.26
	Applied medical science	45	43.2	59	56.7		
	Total	69	39.9	104	60.1		

* *P* ≤ 0.001 is highly significant.

The main indications for SM included menstrual problems (*N* = 143, 82.7 %; *X*^2^, 4.26; *P* =. 04), followed by headache (*N* = 138, 79.8%; *X*^2^, 3.8, *P*=. 05), fever (*N* = 126, 72.8 %; *X*^2^, 5.5, *P* =0.02), pain (*N* = 123, 71.1 %; *X*^2^, 4.41, *P* =0.04), stress (*N* = 61, 35.3 %; *X*^2^, 7.3, *P*=. 007) showed statistically significant among the participants. When compared, applied medical science students (*N* = 45, 43.3%) showed more stress than medical students (*N* = 16, 23.2%). On the contrary, indicators for SM such as fever (82.6%), pain (79.7%), headache (72.5%), and cough flu (58%) were more prevalent among medical students compared to applied medical students (Table 3).

**Table 3:** Indications for self-medication (*N* = 173).

**Indications**	**Category**	**Yes**		**No**		***X^2^***	**P-value**
		**No**.	**%**	**No**.	**%**		
Fever	Medicine	57	82.6	12	17.4	5.5	0.02^*^
	Applied medical science	69	66.3	35	33.7		
	Total	126	72.8	47	27.2		
Pain	Medicine	55	79.7	14	20.3	4.14	0.04^*^
	Applied medical science	68	65.4	36	34.6		
	Total	123	71.1	50	28.9		
Headache	Medicine	50	72.5	19	27.5	3.8	0.05^*^
	Applied medical science	88	84.6	16	15.4		
	Total	138	79.8	35	20.2		
Cough and flu	Medicine	40	58.0	29	42.0	0.82	0.37
	Applied medical science	53	51.0	51	49.0		
	Total	93	53.8	80	46.2		
GIT problems	Medicine	16	23.2	53	76.8	0.03	0.87
	Applied medical science	23	22.1	81	77.9		
	Total	39	22.5	134	77.5		
Diarrhea or constipation	Medicine	16	23.2	53	76.8	0.0002	1.0
	Applied medical science	24	23.1	80	76.9		
	Total	40	23.1	133	76.9		
Mental problems	Medicine	11	15.9	58	47.8	0.01	0.9
	Applied medical science	16	15.4	88	84.6		
	Total	27	15.6	146	84.4		
Inability to sleep	Medicine	17	24.6	52	75.4	1.6	0.2
	Applied medical science	35	33.7	69	66.3		
	Total	52	30.1	121	69.9		
Stress	Medicine	16	23.2	53	76.8	7.3	0.007^**^
	Applied medical science	45	43.3	59	56.7		
	Total	61	35.3	112	64.7		
Avoid sleep during exams	Medicine	14	20.3	55	79.7	1.95	0.2
	Applied medical science	31	29.8	73	70.2		
	Total	45	26.0	128	74.0		
Skin problems	Medicine	25	36.2	44	63.8	1.04	0.3
	Applied medical science	30	28.8	74	71.2		
	Total	55	31.8	118	68.2		
Ear or eye problems	Medicine	19	27.5	50	72.5	0.66	0.4
	Applied medical science	23	22.1	81	77.8		
	Total	42	24.3	131	75.7		
Allergy	Medicine	12	17.4	57	82.6	0.0002	1.0
	Applied medical science	18	17.3	86	82.7		
	Total	30	17.3	143	82.7		
Menstrual problems	Medicine	52	75.4	17	24.6	4.26	0.04^*^
	Applied medical science	91	87.5	13	12.5		
	Total	143	82.7	30	17.3		

* *P* ≤ 0.05 is significant,

** *P* ≤ 0.001 is highly significant.

As shown in Table 4, the most common types of drugs used for SM among participants included antipyretics and analgesics, followed by antispasmodics, antibiotics, antacids, multivitamins, and dietary supplements showed statistically significant.

**Table 4:** Medications used for self-medication (*N* = 173).

**Medicines**	**Category**	**Yes**		**No**		***X^2^***	***P*** **-value**
		**No**.	**%**	**No**.	**%**		
Antipyretic analgesics	Medicine	53	76.8	16	23.2	5.0	0.03^*^
	Applied medical science	93	89.4	11	10.6		
	Total	146	84.4	27	12.1		
Antibiotics	Medicine	39	56.5	30	43.5	8.04	0.005^**^
	Applied medical science	80	76.9	24	23.1		
	Total	119	68.8	54	31.2		
Antihistamine	Medicine	17	24.6	52	75.4	2.9	0.09
	Applied medical science	15	14.4	89	85.6		
	Total	32	18.5	141	81.5		
Antacids	Medicine	27	39.1	42	60.9	44.8	< 0.0001^**^
	Applied medical science	91	87.5	13	12.5		
	Total	118	68.2	55	31.8		
Topical ointment	Medicine	40	23.1	29	16.8	0.4	0.5
	Applied medical science	55	31.8	49	28.3		
	Total	95	54.9	78	45.1		
Multivitamins	Medicine	40	58.0	29	42.0	3.7	0.05^*^
	Applied medical science	75	72.1	29	27.9		
	Total	115	66.5	58	33.5		
Antiemetics	Medicine	7	10.1	62	89.9	0.01	0.93
	Applied medical science	11	10.5	93	89.4		
	Total	18	10.4	155	89.6		
Antispasmodic	Medicine	45	65.2	24	34.8	4.0	0.05^*^
	Applied medical science	82	78.9	22	21.2		
	Total	127	73.4	46	26.5		
Sedative	Medicine	7	10.1	62	89.9	1.1	0.29
	Applied medical science	6	5.8	98	94.2		
	Total	13	7.5	160	92.5		
Anxiolytics	Medicine	5	7.2	64	92.7	0.97	0.32
	Applied medical science	4	3.8	100	96.2		
	Total	9	5.2	164	94.8		
Antidepressant	Medicine	4	5.8	65	94.2	1.9	0.17
	Applied medical science	2	1.9	102	98.1		
	Total	6	3.5	167	96.5		
Skin care	Medicine	51	73.9	18	26.1	0.015	0.90
	Applied medical science	76	73.1	28	26.9		
	Total	127	73.4	46	26.6		
Ear and eye drop	Medicine	46	66.7	23	33.3	3.7	0.06
	Applied medical science	54	51.9	50	48.1		
	Total	100	57.8	73	42.2		

* *P* ≤ 0.05 is significant,

** *P* ≤ 0.001 is highly significant.

As shown in Table 4, the most common types of drugs used for SM among the participants were antipyretics and analgesics (*N* = 146;84.4 %; *X*^2^, 5.0, *P* =0.03) followed by antispasmodics (*N* = 127,73.2%; *X*^2^, 4.0, *P* = 0.05), antibiotics (*N* = 119;68.8 %; *X*^2^, 8.04, *P* = 0.005), antacids (*N* = 118;68.2 %; *X*^2^, 44.8 *P* < 0.00001), multivitamins, and dietary supplements (*N* = 115,66.5 %; *X*^2^, *3.7, p* = 0.05)showed statistical significance. From Table 4, it is evident that applied medical students use more drugs compared to medical students. Also, it was observed that the drugs which are used for mental wellbeing such as sedatives, anxiolytics, and antidepressants were the least consumed. This may be correlated that mental-related drugs are being strictly dispensed in Saudi Arabia.

In the current study, it was observed that family members were the main source of knowledge and motivation for SM (*N* = 116,67.1 %; *X*^2^, 4.3, *P* = 0.038), followed by previous prescription (*N* = 115,66%; *X*^2^, 33.5, *P* = 0.48), self-acquired knowledge (*N* = 112, 64.7 %; *X*^2^, 9.2, *P* = 0.002), consulting pharmacist (*N* = 98,56.7 %; *X*^2^, 16.8, p <0.0001), social media (*N* = 96,55.5 %; *X*^2^, 32.7, *p* <0.0001), showing statistically significant (Table 5). As information sources for SM, applied medical students were found to consult pharmacists (*N* = 77, 74%) and family members (*N* = 76, 73.1%) more frequently than medical students (*N* = 29,42%) and (*N* = 40,58%), respectively. By contrast, medical students relied more on their own knowledge (*N* = 54, 78.9%) than applied medical students (*N* = 58, 55.8%), and they used previous prescriptions more frequently (48, 69.6%) than students of applied medical science (67, 64.4%) (Table 5).

**Table 5:** Source of knowledge for self-medication (*N* = 173).

**Information**	**Category**	**Yes**		**No**		***X^2^***	***P-value***
		**No**.	**%**	**No**.	**%**		
Self-acquired	Medicine	54	78.3	15	21.7	9.2	0.002^**^
	Applied medical science	58	55.8	46	44.2		
	Total	112	64.7	61	35.2		
Consult Pharma source	Medicine	29	42.0	40	58.0	17.9	< 0.0001^**^
	Applied medical science	77	74.0	27	26.0		
	Total	106	61.3	67	38.8		
Previous prescription	Medicine	48	69.6	21	30.4	0.5	0.48
	Applied medical science	67	64.4	37	35.6		
	Total	115	66.5	58	33.5		
Family	Medicine	40	58.0	29	42.0	4.3	0.038^*^
	Applied medical science	76	73.1	28	26.9		
	Total	116	67.1	57	32.9		
Friends	Medicine	21	30.4	48	69.6	0.03	0.86
	Applied medical science	33	31.7	71	68.3
	Total	54	31.2	119	68.8
Social	Medicine	20	29	49	71.0	32.7	< 0.0001^**^
	Applied medical science	76	73.1	28	26.9
	Total	96	55.5	77	44.5

* *P* ≤ 0.05 is significant,

** *P* ≤ 0.001 is highly significant.

It was documented that on getting the adverse effects for SM both groups (medical=55, 79.7% and applied medical science *N* = 92, 88.5%) consulted the physician or doctor (*N* = 147, 85%; *X*^2^, 2.49, *p* = 0.11). The statistically high significant observation was made among participants in consultation with the pharmacist for the remedy of the adverse reaction (*N* = 98, 56.7 %; *X*^2^, 16.8, *P* < 0.0001) followed by a switch to other drugs (*N* = 68, 39.3 %; *X*^2^, 7.97, p =0.005).

The majority of students documented that on getting adverse effects for SM, they would neither increase (*N* = 168, 97.1%); nor decrease (*N* = 151, 87.3 %), the dosage of the drugs (Table 6).

**Table 6:** Alternative plan if adverse effect of SM (*N* = 173).

**Alternative plan**	**Category**	**Yes**		**No**		***X^2^***	***P*** **-value**
		**No**.	**%**	**No**.	**%**		
Consult doctor	Medicine	55	79.7	14	20.3	2.49	0.11
	Applied medical science	92	88.5	12	11.5		
	Total	147	85.0	26	15.0		
Consult Pharma	Medicine	26	69.2	43	62.3	16.8	0.00004^**^
	Applied medical science	72	41.6	32	41.3		
	Total	98	56.7	75	43.4		
Increase dosage	Medicine	3	4.3	66	95.7	<5 unit	
	Applied medical science	2	1.9	102	98.1		
	Total	5	2.9	168	97.1		
Decrease dosage	Medicine	12	17.4	57	82.6	2.3	0.13
	Applied medical science	10	9.6	94	90.4		
	Total	22	12.7	151	87.3		
Switch to other drug	Medicine	36	52.2	33	47.8	7.97	0.005^**^
	Applied medical science	32	30.8	72	69.2		
	Total	68	39.3	105	60.7		

** *P* ≤ 0.001 is highly significant.

It is found that there is a strong correlation among variables for the causes of self-medication. The time saving was highly correlated with self-confidence and quick relief of minor diseases (Table 7).

**Table 7:** Correlations (r) between the variables of reasons (*N* = 173).

	**A**	**B**	**C**	**D**	**E**	**F**	**G**	**H**	**I**	**J**
A	1	0.165^*^	0.213^**^	0.232^**^	0.197^**^	0.145	0.193^*^	0.351^**^	0.119	0.090
B		1	0.131	0.116	0.187^*^	0.106	−0.165^*^	0.081	0.386^**^	0.152^*^
C			1	0.262^**^	0.019	0.037	0.256^**^	0.178^*^	0.090	0.106
D				1	0.292^**^	0.228^**^	0.151^*^	0.179^*^	0.181^*^	0.113
E					1	0.358^**^	0.163^*^	0.197^**^	0.163^*^	0.206^**^
F						1	0.298^**^	0.133	0.098	0.270^**^
G							1	0.255^**^	−0.031	0.226^**^
H								1	0.133	0.064
I									1	0.007
J										1

2-tailed:

* Correlation is significant at the 0.05 level.

** Correlation is highly significant at the 0.01 level.

A, Time saving; E, Previous experience; I, Laziness to see the doctor.

B, Illness as minor; F, Availability of drugs; J, Use leftover drugs.

C, Economical; G, Free sample.

D, Quick relief; H, Self confidence.

## 4. Discussion

The present study revealed that 80.84% (*N* = 173) of participants (medical 84.15 %, *N* = 69) and applied medical science (78.79%, *N* = 104) reported SM practiced 3 months prior to the study. However, several studies reported different prevalence rates ranging from 55 to 98% (5, 6, 12, 13). Irrational use of medicines has been shown to be most prevalent among university students and is a key issue for the WHO in its efforts to promote the rational use of medicines. Instinctively treating one's illness has evolved into a common human rights practice as SM (7). Younger generations presently have more convenient access to medicines than in the past, which could be harmful to their health, especially if they use inappropriate and ineffective medicines (8).

The main motivation for SM included a quasi-health issue, the urge for alleviating the symptoms, and the means to escape waiting hours at clinics (9).

### 4.1. Reasons for self-medication

The study revealed that the main reason for SM was time-saving among the participants (71.7%), followed by minor illness (71.1%), previous experience (65.9%), quick relief from ailments (59.0%), self-confidence (54.3%), laziness to consult the physician (50.9%), and the last reason was to use of leftover drugs (37.6%). Compared to applied medical science students, the prevalence of time-saving (84.1%), considering disease as a minor illness (76.8%), self-confidence (68.1%), and laziness to consult the physician (68.1%), was reported higher among medical college students. On the contrary, the prevalence of quick relief from ailments (82.7%) and the use of leftover drugs at home (43.2%) were common among applied medical science students. Students believed that it was convenient and easy to choose their medications on their own for mild illnesses for faster relief than visiting the physician. Other factors that may lead to SM were economic condition, previous experience, availability of medications, self-confidence, and laziness to consult the physician.

A statistical significant correlation between time saving, economical, minor illness, quick relief, previous experience, and self-confidence was observed in the present study. These findings are consistent with those of other studies which also mentioned time savings by not visiting the physician (13–16).

The high prevalence of SM among undergraduate students with a health science background may be attributed to the perception that they have sufficient knowledge and that an illness with mild symptoms will not have any serious effects. In the present study, the uses of leftover medications were more prevalent among the present study participants. A similar study in Nepal by Manandhar Shrestha et al. revealed that leftover medicines use was 22.6% as compared to the present study (39.9%), it was lower prevalent (17).

Leftover medication storage could promote irrational medication use. In comparison to nations with strict drug regulations, developing nations are more likely to encourage SM by using leftover medication due to economic factors.

Peer guidance and easy access to drugs were both considered to have an impact on SM in the present study. The causes of SM reported in this study were similar to those found in earlier studies, including prior knowledge, experience, recommendations, and guidance from pharmacists, friends, and family. The present survey results were consistent with research reported in Madinah, Saudi Arabia, by Alshahrani et al. (18).

### 4.2. Indications for self-medication

Most of the participants in the survey revealed that they self-medicated for minor illnesses. Menstrual problems were the highest reported in the present study followed by headache, fever, pain, cough, and flu. Mental-related issues were the least prevalent in the present study. These findings were consistent with other studies (10, 11, 15, 19, 20). Prudent SM, particularly for minor and chronic health issues, may be beneficial to individuals as well as healthcare systems (21). Previous studies revealed SM was used to treat ailments, common of which were symptoms of the common cold such as aches and pains, nasal congestion, and seasonal allergies. These observations are consistent with the present study findings. Other studies considered indications of SM such as constipation, nausea, insomnia, fatigue, and skin rash ranging from 23 to 9.48% consistent with the present study (13, 17, 19, 21).

### 4.3. Sources of drug information

In the present study, family members, previous prescriptions, and self-acquired knowledge were the main sources of SM. Self-acquired knowledge and previous prescription were highest among the medical students as compared to applied medical science students followed by the use of social media, and the least was advice from friends. While consulting the pharmacist, followed by motivation from social media and family was most common among the applied medical students as compared to medical students. The majority of the participants in the present study assumed that they have adequate knowledge of medications and diseases. These findings are consistent with a similar study conducted in Kerman by Zardosht et al., which indicated that students' knowledge about diseases and their treatment can persuade the use of SM (8, 11, 22).

There was also a strong agreement that pharmacists played a pivotal role in motivating the SM. Similar results were observed among University students from Saudi Arabia, Kuwait, and Bahrain (13, 17, 23).

### 4.4. Commonly used drugs

In the present study, compared to medical students, applied medical science students used more analgesics, antibiotics, antispasmodics, topical creams, and nutritional supplements.

Health science students are more prone to SM with analgesics; they may be at risk of analgesic overuse (21). The present study revealed antispasmodics followed by analgesics and antipyretics were the most frequently used drugs for SM by participants to treat minor illnesses such as fever and headaches. Surveys carried out in India, Pakistan, Bangladesh, Ethiopia, and Iran also revealed similar findings pertaining to the consumption of analgesics and antipyretics (2, 8, 11, 14, 20, 24, 25). As compared to studies conducted in Croatia 38% and Saudi Arabia 30%, the findings of the present study show significantly higher consumption of antibiotics as SM (68.8%) (3, 6, 18).

Data from the present study (analgesics and antipyretics 84.4%; antibiotics 68.8%) were higher than Okyay and Erdogan's study in Turkey, which observed that analgesics and antibiotics were the most commonly consumed medicines by students without a prescription, accounting for 39.5 and 36.9%, respectively (19).

There are different potential risks of SM such as incorrect self-diagnosis, administration, or dosing. According to the WHO in developing countries, the major cause of antibiotic resistance is the SM was an improper dose of antibiotics that might lead to various detrimental effects, including the sensitivity of antibiotics to microbial flora, development of multidrug resistance to pathogens, and other related symptoms (10, 11, 26–29). The SM of antibiotics needs to be stopped and strictly monitored by the regulatory authority (11, 29).

### 4.5. Attitude and response toward adverse effects of drugs

Approximately 85.0% of the students in the present study indicated that they would seek medical advice from a physician (88.5%), followed by consultation with a pharmacist (56.7%, *P* < 0.0001) if they get the adverse effect of drugs. Klemenc-Ketis et al. and Alam et al. reported similar results when they reported that medical students consulted doctors and pharmacists about the adverse effects of drugs (6, 10, 15, 21). The students practice SM at first, but if it does not work, they usually prefer to go for medical consultation (21). In accordance with other studies, the attitude toward SM was positive, meaning students thought that it was not good to self-medicate (2, 11, 13). Reasons not to go for SM were risks of adverse effects, using the wrong medication, drug interaction, misdiagnosis, and drug abuse and dependence (10, 30). The adverse effects of such practices needed to be emphasized in the community and steps to constrain it. The rampant irrational use of antimicrobials, without medical consultation, may result in a high probability of missed diagnosis or delays in appropriate treatment, and increased morbidity (9, 31).

### 4.6. Limitation of the study

The research has some limitations of its own. Because it was only conducted among female students, the study did not compare the two genders. The survey being a self-reported survey could have led to insufficient or excessive reporting of self-medication practices. Some students did not show interest in the study despite efforts to encourage them, possibly, which may have an impact on the sample size. Finally, the focus of the questionnaire was mainly on allopathic medications. The questionnaire did not include any conventional medications.

## 5. Conclusion

In this study, it was observed that the most common reason for self-medication was to get quick relief from the disease and the students thought it would be a waste of time to visit the doctor and took the illness non-serious. The most common drugs used were antispasmodics followed by analgesics and antipyretics.

In this study, it was observed that the most common reason for self-medication was to get quick relief from the disease and the students thought it would be waste of time to visit the doctor and took the illness non-serious. The most common drugs used were antispasmodic followed by analgesic and antipyretics. The most common indications for SM reported were menstrual problems, fever, and headache. The family members were the main sources to motivate SM followed by self-acquired knowledge. When getting adverse effects most of them consulted doctors. It would be suggested that regulations and monitoring of drug dispensing should be strengthened by the legislative authorities.

## 6. Recommendations

We recommend developing a healthcare system to incorporate the dispensing and sale of medicines more efficiently. Provide health information and create awareness of SM, their benefits and adverse effects, and their impact on health in the long run among the young generation. Social media such as television or public platforms can be used to create awareness in the community. Finally, the government must enforce strict rules and regulations mandating pharmacists to only dispense prescription medications.

## Data availability statement

The raw data supporting the conclusions of this article will be made available by the authors, without undue reservation.

## Ethics statement

The studies involving human participants were reviewed and approved by Institutional Review Board of Majmaah University (MUREC-Dec.15/COM-2020/13-1). Majmaah University, Saudi Arabia. The patients/participants provided their written informed consent to participate in this study.

## Author contributions

Conceptualization: SL and RE. Methodology: SL and HA. Validation and supervision: SL. Formal analysis and data curation: MK and SL. Data collection: MAlh. Writing—original draft preparation: SL, RK, and ZA-E. Writing—review and editing: SL, MAlz, HA, and RE. Visualization: MK. Project administration: RE. All authors have read and agreed to the published version of the manuscript.
